# The Cologne-Mecklenburg-Vorpommern DMEK Donor Study (COMEDOS) — design and review of the influence of donor characteristics on Descemet membrane endothelial keratoplasty (DMEK) outcome

**DOI:** 10.1007/s00417-022-05594-w

**Published:** 2022-03-16

**Authors:** Silvia Schrittenlocher, Mario Matthaei, Björn Bachmann, Claus Cursiefen

**Affiliations:** 1 Faculty of Medicine and University Hospital Cologne, Department of Ophthalmology, University of Cologne, Kerpener Str. 62, 50937 Cologne, Germany; 2 Center for Molecular Medicine Cologne (CMMC), University of Cologne, Cologne, Germany

**Keywords:** DMEK, Lamellar keratoplasty, Cornea, Donor, Graft preparation

## Abstract

**Background:**

Posterior lamellar keratoplasty and especially Descemet membrane endothelial keratoplasty (DMEK) are gaining interest worldwide. Little is known about the influence of donor factors on DMEK outcome. Here we provide an overview of the existing peer-reviewed literature on this topic and present the design of the upcoming cooperation study COMEDOS (Cologne-Mecklenburg-Vorpommern DMEK Donor Study).

**Methods:**

A literature search of PubMed and MEDLINE was conducted to retrieve articles published between September 2013 and May 2021. Seventeen peer-reviewed articles were selected. Design and concept of the prospective COMEDOS are outlined.

**Results:**

Main interest parameters were the donor diabetes mellitus status, age, and lens status. There is a large heterogeneity regarding the sample size, study design, and investigated parameters. There seems to be a consensus that younger donors are associated with tighter rolls, a more difficult preparation, and unfolding setting. Diabetic donors seem to increase the risk of tissue tearing due to adherences and result more frequently in preparation failure. The COMEDOS aims not only to analyze the diabetes status of the donor, but also to correlate all donor systemic comorbidities and their ophthalmologic history to the DMEK clinical outcome. Furthermore, a correlation of Descemet membrane lamella preparation and surgery outcome is planned.

**Conclusion:**

Currently, there is a lack of knowledge regarding the effect and impact of donor tissue characteristics on DMEK outcome and complications. An in-depth investigation is planned by the upcoming COMEDOS to close this knowledge gap.
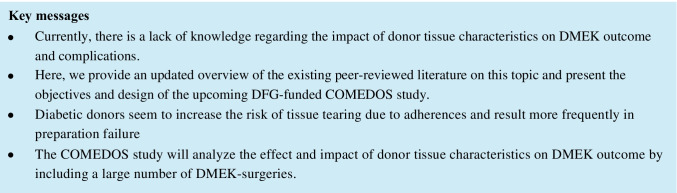

## Introduction

The field of corneal transplantation has been revolutionized during the recent years by introducing Descemet membrane endothelial keratoplasty (DMEK). It has become the procedure of choice for the treatment of corneal endothelial diseases such as Fuchs endothelial dystrophy in many countries [1–4]. This procedure allows rapid visual recovery and has fewer immunological graft rejections compared to conventional penetrating keratoplasty (PK) [1, 5, 6]. However, the learning curve of this surgical option is longer due to difficulties of graft preparation, unfolding, and unfolding behavior. Difficulties of graft preparation include tears, splitting, and rolling and may lead to discarding of the tissue, interruption of the surgery, and, eventually, financial loss. Since Descemet membrane (DM) has a thickness of 10 to 15 µm, tears in the membrane can easily occur while stripping. There is mounting evidence that the properties of DM stripping correlate with donor factors. The DMs of different donors vary in properties regarding their fragility or adhesion to the corneal stroma and may therefore influence the success of donor preparation. DM consists of two layers that can separate during stripping, so-called splitting of the membrane. It has been reported that diabetic donor corneas are more prone to DM splitting and can present difficulties during graft preparation [7]. Retained remnants of DM during separation seem to be a cause for graft detachment, the most common postoperative complication after DMEK surgery.

Furthermore, rolling characteristics are very different in different donor ages. Besides properties of the recipients’ eye, the tendency of DM to form a roll is the most important factor in determining how atraumatic the unfolding of the graft can be performed inside the anterior chamber. Schaub et al. reported that donor age can play a role in the graft preparation and the unfolding behavior of the graft lamella during surgery; donor tissue from elderly patients (above 70 years of age) tends to roll less than tissue obtained from younger donors (below 50 years of age) [8–10]. Also, it has been postulated that donor lens status influences the outcome of the DMEK surgery [11]. Another interesting aspect is the role of the culture media on endothelial cell viability and its effect on the actual scroll width during surgery [12]. However, the previous studies include either a small number of eyes or only present short-term results.

Overall, there is a lack of knowledge about the long-term effect and impact of donor tissue characteristics on DMEK outcome and complications [10, 11]. This is in marked contrast to the conventional PK or Descemet’s stripping automated endothelial keratoplasty (DSAEK). Several large studies investigated the impact of donor tissue characteristics on outcome of penetrating keratoplasty [13–16] and DSAEK [17–19]. The Cornea Donor Study demonstrated that both old and younger donors are suitable leading to wider eligibility criteria for corneal transplantation [20, 21]. The Specular Microscopy Ancillary Study examined the effect of donor age and other perioperative factors on long-term endothelial cell loss after penetrating keratoplasty (PK) and found that substantial cell loss occurs on the long term after PK, with the rate of cell loss being slightly higher with older donor age [21]. Terry et al. found an association between diabetes and DSAEK outcome and complications [22]. These studies provided evidence and guideline support for corneal surgeons by assessing the suitability of the donor tissue.

Nevertheless, studies with large sample sizes with meaningful follow-up and solid long-term assessments of donor characteristics on clinical outcome are still lacking in the field of DMEK. This knowledge gap has an important impact on clinical work as it could influence the prediction of surgery outcome. By considering preexisting donor conditions and correlating these with graft characteristics, the outcome of the DMEK surgeries could be positively influenced and complications could be prevented. In times of donor shortage, preventing tissue discarding because of graft preparation complications is also a key issue.

In this context, the objectives of this article are (i) to review the currently existing peer-reviewed literature on this topic and (ii) to present the objectives and design of the upcoming COMEDOS which aims to correlate donor characteristics and DMEK outcome.

## Patients and methods

### Search method

A search of electronic databases was conducted to retrieve articles published between September 2013 and May 2021 in PubMED and MEDLINE. Search term combination used were as follows: “DMEK” or “Descemet Membrane” AND “donor” OR “graft” OR “preparation.” Only publications which matched the search terms in the same context were included. Publications in English or German were included; other languages were excluded. All types of research (observational study, cohort study, clinical trial) were included. A literature management software (EndNote X9.1, Thomson ResearchSoft, Thomson Corporation, Stanford, CT) was used to manage the records.

### Study selection

Only studies on humans were selected. Only studies reporting on the influence of donor tissue characteristics on DMEK were selected.

### Data collection

A spreadsheet software (MS Excel Version 16.50; Microsoft, Seattle, WA) was used for standardized data extraction. Data recorded per publication included as follows: country and region of data collection, number of cases by indications, year of publication, study setting (single center, multicenter, eye bank, or transplant register), study design (retrospective, prospective, and consecutive, selective, randomized), and diagnostic base (clinical or histopathological diagnosis).

### Cologne DMEK database

The *Cologne DMEK database* was established in 2015 and includes all data related to DMEK surgeries performed in the Department of Ophthalmology, University Hospital of Cologne. Since 2011, more than 4000 DMEK surgeries have been performed at the Department of Ophthalmology in Cologne. Our DMEK database includes records of more than 3000 patients with a minimum follow-up of 1 year. The clinical information contained is demographics, medical history, preoperative status of the eye, details of surgery, details of donor tissue, and postoperative outcomes (visual acuity, intraocular pressure, endothelial cell count, corneal topography, eye imaging, graft status, complications). There are no exclusion criteria. The prospective *Cologne DMEK database* is filled retrospectively through the existing data collection systems. All medical data is handled confidentially according to the “Good Epidemiological Practice (GEP)” guideline and with ethics committee approval by the Ethics Commission of Cologne University’s Faculty of Medicine (14–373). Pseudonym using consecutive numbering is being used without patient abbreviation or birthdate. Only designated investigators can access the REDCap data collection program. The development of the database provides a broad clinical data base and an easy access for future clinical studies in this field on reporting the long-term outcomes and safety of this procedure [23–25].

## Results

A total of 353 articles matched the search terms. After filtering and screening for relevance by title and abstract, 17 articles were found suitable to include in the final review (Table 1). All types of research were included (observational studies, cohort studies, clinical trials). Sixteen out of 17 were single-center studies and 1 was a multicenter study. Seven studies were conducted in Germany, 9 in the USA, and one in the Netherlands. The cohort amount varied between 26 and 1748 eyes. 10/17 listed donor age as the main interest parameter, 5/17 donor diabetes status, and 2/17 donor lens status. 3/17 described experimental techniques while 14/17 focused on the clinical outcome. In the 14 clinical studies, the study design was retrospective. The data collection was consecutive in all studies and they were published between 2013 and 2020.

**Table 1:** Included studies reporting the influence of donor characteristics on DMEK outcome

	Author	Year of publication	Country	Cases	Study setting	Study design	Data collection	Primary diagnostic base	Secondary diagnostic base	Donor tissue preparation	Conclusion
1	Straiko et al	2020	US	857	Single center	Retrospective	Consecutive	Donor age	Preoperative ECD, preservation time, death-to-preservation time, preparation-to-surgery time, donor diabetes status, rebubbling, 6-mo ECL	Eye bank prepared	Donor age, donor ECD, and preservation time had no significant effect on the rate of rebubbling
2	Hill et al	2020	US	661	Single center	Retrospective	Consecutive	Donor age	Scrolling, rebubbling, 3 and 6 mo ECL	Eye bank prepared	No significant difference between younger and older donors
3	Schaub et al	2020	D	1748	Single center	Retrospective	Consecutive	Donor age	BSCVA, ECD, and CCT at 3 and 6 mo and at 1, 2, and 3y	Shortly before surgery	Older donors, ≥ 80 to 94 years of age, seem to produce comparable mid-term functional results following DMEK surgery compared to younger donors
4	Lapp et al	2018	D	1055	Single center	Retrospective	Consecutive	Donor lens status (pseudophakia)	Graft survival, rebubbling rates, ECL	Shortly before surgery	Grafts from pseudophakic donors can be safely prepared and used for DMEK, leading to comparable graft survival rates and ECL and a reduced rebubbling rate
5	Price et al	2017	US	1791	Single center	Retrospective	Consecutive	Donor diabetes	4-y graft survival, ECL	2d ahead preparation	More difficult preparation in donors with diabetes but no significant difference regarding 4-year survival, rebubbling rate, and ECL
6	Schaub et al	2017	D	181	Single center	Retrospective	Consecutive	Donor lens status	BCVA, ECD, CCT, rebubbling	Shortly before surgery	Phakic donors have higher ECD, comparable results
7	Williams et al	2016	US	125	Single center	Retrospective	Consecutive	Donor diabetes	5 points — risk stratifications rating scale	Eye bank prepared	Diabetes rating scale stratifies the risk of preparation failure
8	Schwarz et al	2016	US	37	Single center	Retrospective	Consecutive	Donor diabetes	Experimental technique for quantifying the force required to separate the endothelium-Descemet membrane complex (EDM) from stroma and differences in adhesion strength between diabetic and non-diabetic donor corneas	Shortly before surgery	Chronic hyperglycemia from diabetes mellitus results in a phenotypically more adhesive interface between Descemet membrane and the posterior stroma in donor corneal tissue
9	Rodriguez-Calvo de Mora et al	2016	NL	500	Single center	Retrospective	Consecutive	Donor age	Storage time, ECD	n.s	Older donors and short storage time are better suited
10	Schaub et al	2016	D	529	Single center	Retrospective	Consecutive	Donor age	BCVA, ECD, CCT, rebubbling	Shortly before surgery	Young donors up to 17 years are suitable
11	Bennett et al	2015	US	26	Single center	Blinded	Consecutive	Donor age	Scrolling scale, diabetes	Shortly before surgery	Using older donors reduces EDM scroll tightness
12	Vianna et al	2015	US	563	Single center	Retrospective	Consecutive	Donor diabetes	Hypertension, obesity, hyperlipidemia, 2 groups DMEK preparation success and DMEK preparation failure	Eye bank prepared	Tissues from donors with diabetes mellitus (especially with longer disease duration) and hyperlipidemia or obesity were associated with higher failure rates in DMEK preparation
13	Maier et al	2015	D	169	Single center	Retrospective	Consecutive	Donor age	Gender, ECC, storage time	Eye bank prepared	No correlation between corneal donor tissue characteristics and the degree of difficulty of unfolding using graft lamella older than 49 years
14	Gorovoy et al	2014	US	116	Single center	Retrospective	Consecutive	Donor age	Gender, postmortem tissue age, contralateral eye data, peeling time	Eye bank prepared	Grafts from donors in which there was a complication should be excluded for the second eye A complication in the preparation of the first eye such as a very long peel time or a graft tear should be excluded for DMEK in the second eye
15	Greiner et al	2014	US	359	Multicenter	Retrospective	Consecutive	Donor diabetes	Graft preparation failure	Eye bank prepared	Diabetes increases the risk of preparation failure ninefold
16	Heinzelmann et al	2014	D	31	Single center	In vitro	Consecutive	Donor age	ECD, width roll, double-bubble unscrolling technique	Shortly before surgery	Preferably older donors with high endothelial cell densities for DMEK
17	Schlötzer-Schrehardt et al	2013	D	350	Single center	Retrospective	Consecutive	Donor age	EM, immunhistology	Shortly before surgery	Manual preparation of grafts for DMEK with reproducible tissue qualities is possible in the vast majority (98%) of donor corneas

Abbreviations: *ECD*, endothelial cell density; *ECL*, endothelial cell loss; *EM*, electron microscopy; *D*, Germany; *US*, United States; *NL*, Netherlands; *BSCVA*, best spectacle-corrected visual acuity; *CCT*, central corneal thickness; *DMEK*, Descemet membrane endothelial keratoplasty; *n.s.*, not specified; *d*, day; *mo*, month; *y*, year

The donor tissue preparation in all German studies was carried out shortly before transplantation, while in the US report, the tissue was previously prepared in the eye bank. The preparation time in the Dutch report was not specified.

### Age

10/17 publication considered the donor age a primary factor [26–35]. There seemed to be a consensus that both younger and older donors were suitable and had a similar clinical outcome. However, grafts from younger tissues had an increased tendency of curling and rolling while grafts from older donors tended to roll less [31]. This can be favorable in using tissue from older donors as the unfolding takes less time. Gorovoy et al. suggested that the second eye of donors with consecutive complicated preparation should be excluded [33].

### Diabetes

Tissues from diabetic donors are more difficult to process and are associated with a higher rate of graft failure preparation. Greiner et al. postulated a ninefold increased risk of graft preparation in diabetic donors [7]. However, no detailed information about the diabetes status concerning diabetes type, treatment, etc. was available in the previously cited study. Williams et al. developed a 5-point rating scale for risk stratification in diabetic donors. Factors like diabetes duration, obesity, body mass index, insulin treatment, and hypertension increased the risk of preparation failure [36].

### Lens status

Two publications analyzed the clinical outcome of phakic compared to pseudophakic donors [11, 37]. Phakic donors have higher endothelial cell densities (ECD) but both types of donors have comparable graft survival rates and endothelial cell losses (ECL) and can be safely used for DMEK [11, 37].

### Descemet membrane preparation

Seven publications described the preparation difficulties of the DM lamella [7, 26, 31, 33–35, 38]. Scrolling behavior, peeling time, with roll, and graft preparation failure were analyzed. These properties were correlated to donor age and diabetes. The consensus is that tissue from older donors scroll less and present with a larger width of the roll. Schlötzer-Schrehardt et al. demonstrated by means of electron microscopy and immunohistochemistry that manual preparation of grafts for DMEK with reproducible tissue qualities is possible in the vast majority (98%) of donor corneas.

### Objectives and design of COMEDOS

COMEDOS is a DFG funded (SCHR 1666/2–1) collaborative study between the largest German DMEK database and the Multi-Tissue Bank Mecklenburg-Western Pomerania (GBM-V) (https://www.gbm-v.de/) and the Society for Transplantation Medicine Mecklenburg-Vorpommern (GTM-V) (https://www.gtm-v.de/), which will dispose of a large donor history database as well as merge and correlate information of donor characteristics to clinical DMEK outcome.

The upcoming COMEDOS will be a retrospective analysis. However, the data collection in the Cologne DMEK database is prospective. In the following, we would like to outline the study design in detail: several standardized donor-related information (related to donor itself: like cause of death, preexisting systemic diseases, preexisting ocular diseases, preexisting systemic surgeries, preexisting eye surgeries, systemic and ocular medication, smoking behavior etc.; related to donor tissue: donor graft endothelial cell count (ECC), culture time, culture medium, death-to-preservation time, death-to-use time etc.) will be included and correlated with the tissue preparation characteristics of the donor graft (such as unfolding time, degree of difficulty when stripping and peeling the donor graft, grading of rolling behavior, tissue tears, central and peripheral attachments, width of the DMEK roll, staining behavior, overall graft fragility etc.) and the impact on the clinical outcome of the DMEK-surgery.

One aim is to analyze the impact of different donor characteristics (e.g., ECC, gender, race, smoking behavior, diabetes mellitus, lens status, storage time, storage temperature, death-to-preservation time, death-to-use time) on the clinical outcome following DMEK surgery (BSCVA, long-term endothelial cell density, speed of vision recovery, rebubbling rate, macular edema, immune reaction, and rejection events etc.).

Also, we plan to evaluate the role of donor characteristics on the graft behavior during tissue preparation and surgery (unfolding time, stripping, peeling, rolling behavior, tears, central and peripheral attachment, width of roll, staining, overall graft fragility) on the clinical outcome. To achieve this, we plan to collect stripping data in relation to the general condition of the donor tissue from over 2000 donors (general diseases, infectious diseases, dialysis, previous eye surgeries, cardiovascular disease, death cause etc.) and also to perform histological examinations from donor Descemet membrane remnants or discarded tissue.

All donor collection parameters and data regarding the donor graft (such as ECC, culture time, culture medium, death-to-preservation time, death-to-use time) are standardized and meticulously documented by the certified Multi-Tissue Bank Mecklenburg-Western Pomerania (Gewebebank Mecklenburg-Vorpommern gGmbH; www.gbm-v.de).

The preparation characteristics that are collected in our clinical department are also collected and documented in a standardized fashion. There are three DMEK surgeons involved in the analysis (BB, CC, MM). All three surgeons were involved in the documentation of a standardized questionnaire at the end of every surgery. The questionnaire includes graded information about the graft preparation characteristics (such as stripping, central and peripheral attachments, splitting, staining, fragility).

The statistical analyses will be performed in collaboration with the Institute for Medical Statistics and Computational Biology (IMSB), University of Cologne.

The study has been registered in the “German Clinical Trials Register (Deutsches Register Klinischer Studien) — DRKS00028034.”

## Discussion

The current review provides an overview of the literature which analyzed the influence of donor factors like age, diabetes, and lens status on the clinical outcome of DMEK. The study methods and cohort sizes of the existing literature are heterogenous and have several limitations: variation of sample sizes, period of follow-up, and graft preparation variability (shortly before surgery by the surgeon and eye bank preparation ahead) are some. Also, the diabetic status and complication degree of the patient were not defined in the cited studies. As previously reported by Luke et al., the overall quality of data is low and further research on this field is warranted [39].

However, there seems to be a consensus that the preparation of the DM graft is influenced by age and comorbidities of the donor. These factors reflect themselves in the rolling behavior of the graft intraoperatively. Donor age seems to be an important factor regarding the rolling behavior of the graft [31]. Some surgeons take into account the donor age and may request tissue form older donors to use in patients with deep anterior chamber-like aphakic eyes or highly myopic patients. Several studies showed that DM tissues from older donors are easier to handle [31]. Nevertheless, the authors seem to agree that both young and older donors are suitable for DMEK grafting with comparable clinical outcome [10, 28].

Another factor to keep in mind in relation to older donors is the lens status as the prevalence of pseudophakia increases with age. This factor can influence the endothelial cell density (ECD). Lapp et al. reported that pseudophakic eyes are comparable to those with grafts from phakic eyes [37]. This conclusion was previously confirmed by Schaub et al. who reported that pseudophakic transplants with high ECD led to comparable functional results in recipients after a 2-year course [11].

During graft preparation, the separation of the DM from the underlying stroma represents a delicate step. Diabetic donor corneas seem to be more prone to DM splitting and are linked to difficulties during graft preparation [7, 36, 38, 40, 41]. Greiner et al. implicated a molecular alteration and suspected a stronger adhesion and increased tendency for tearing due to glycation products from chronic hyperglycemia and deposit in the interfacial matrix [7]. Schwarz et al. quantified the differences in the peeling process on normal versus diabetic tissue and proved that chronic hyperglycemia from diabetes mellitus results in a phenotypically more adhesive interface between Descemet membrane and the posterior stroma in donor corneal tissue [38].

Currently there are no guidelines of strong evidence on how the DMEK surgeon should choose the donor tissue. This is in contrast to several previous studies in the field of PK and DSAEK.

As postulated by large cohort studies in PK and DSAEK, several donor factors like donor age, gender, and diabetes seem to play a role on the recipient’s clinical outcome [20, 22]. One of these studies was the Cornea Donor Study (CDS) which was designed as a prospective, double-masked, controlled trial to determine the role of donor age in long-term corneal graft survival and it showed that graft survival is similar using corneas from donors ≥ 66.0 years and donors < 66.0 years for PK [20]. Another study, the Specular Microscopy Ancillary Study, showed that a substantial cell loss seems to occur in eyes with a clear graft 10 years after PK, with the rate of cell loss being slightly higher with older donor age [21]. Regarding DSAEK, a Scandinavian study based on the Swedish Cornea Transplant Registry analyzed the effect of donor characteristics on the clinical outcome and found that low donor ECD was not detrimental to graft survival, whereas donor gender seemed to influence the outcome at the end of the 2-year follow-up with male donors being associated with lower 2-year graft survival, but not with rejection rate [18]. In the Cornea Preservation Time Study (CPTS), the 3-year DSAEK outcome was determined. The authors found that DSAEK outcome was influenced by the diabetic status of the donor [22].

Such large studies are necessary for guiding surgeons on the suitability of donor tissue and impact on outcome. However, there is still a lack of knowledge and large studies are missing in the field of DMEK. In our opinion, further research is required to reinforce these data with strong statistical measures and correlation of the effect of other donor characteristics on DMEK graft preparation. Future research should include an evaluation of the impact of pre-existing diseases and previous surgeries of the donor on the clinical outcome and complications. A correlation of a standardized evaluation of the intraoperative graft preparation features on outcome is needed. In addition, the intraoperative graft behavior should be followed-up for a minimum of 2 or 3 years to assess the clinical outcome of DMEK surgery.

## Conclusion

In this review, we demonstrated that there is a huge gap of knowledge regarding the impact of donor tissue characteristics on DMEK outcome and complications [42]. The upcoming COMEDOS study aims to address this unmet need. Not only diabetes but also other comorbidities and ocular diseases will be analyzed and correlated to graft preparation and clinical outcome. The study will provide a comprehensive assessment of donor characteristics and their influence on the clinical outcome of DMEK surgery.
